# Supplementary figures and images for: Dynamics of Pellet Fragmentation and Aggregation in Liquid-Grown Cultures of Streptomyces lividans
Source: Front Microbiol. 2018 May 11;9:943. doi: 10.3389/fmicb.2018.00943 (PMC5958208; doi:10.3389/fmicb.2018.00943)

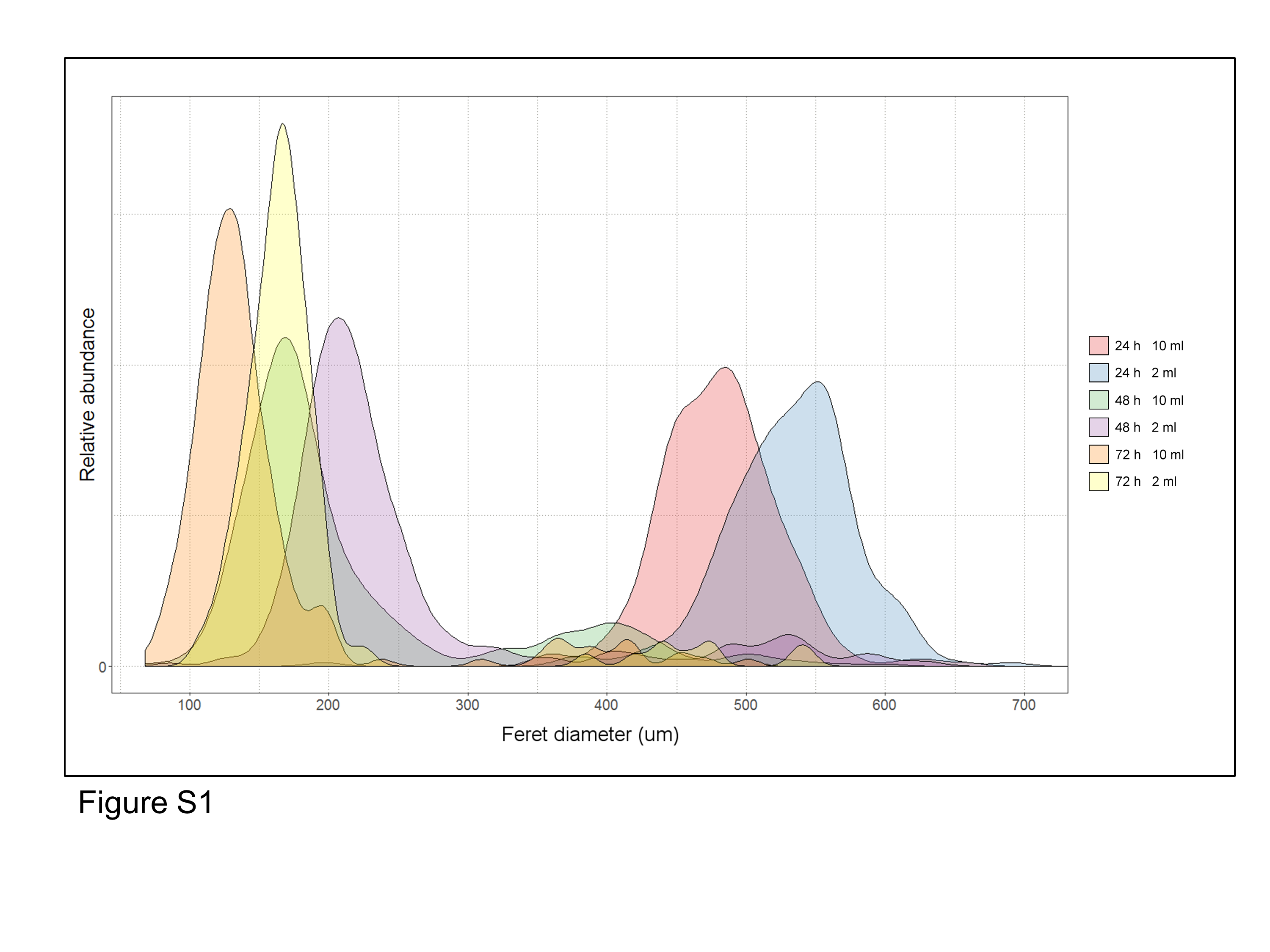

Supplement: FIGURE S1 — Size distributions of pellets in diluted cultures of Streptomyces lividans 66. TSBS cultures were inoculated with 2 or 10 ml of seed cultures that had been grown for 24, 48, or 72 h. The plots represent the size distribution of at least 300 pellets per sample, obtained after 24 h of growth. All sizes are indicated in micrometers. [file Image_1.TIF]

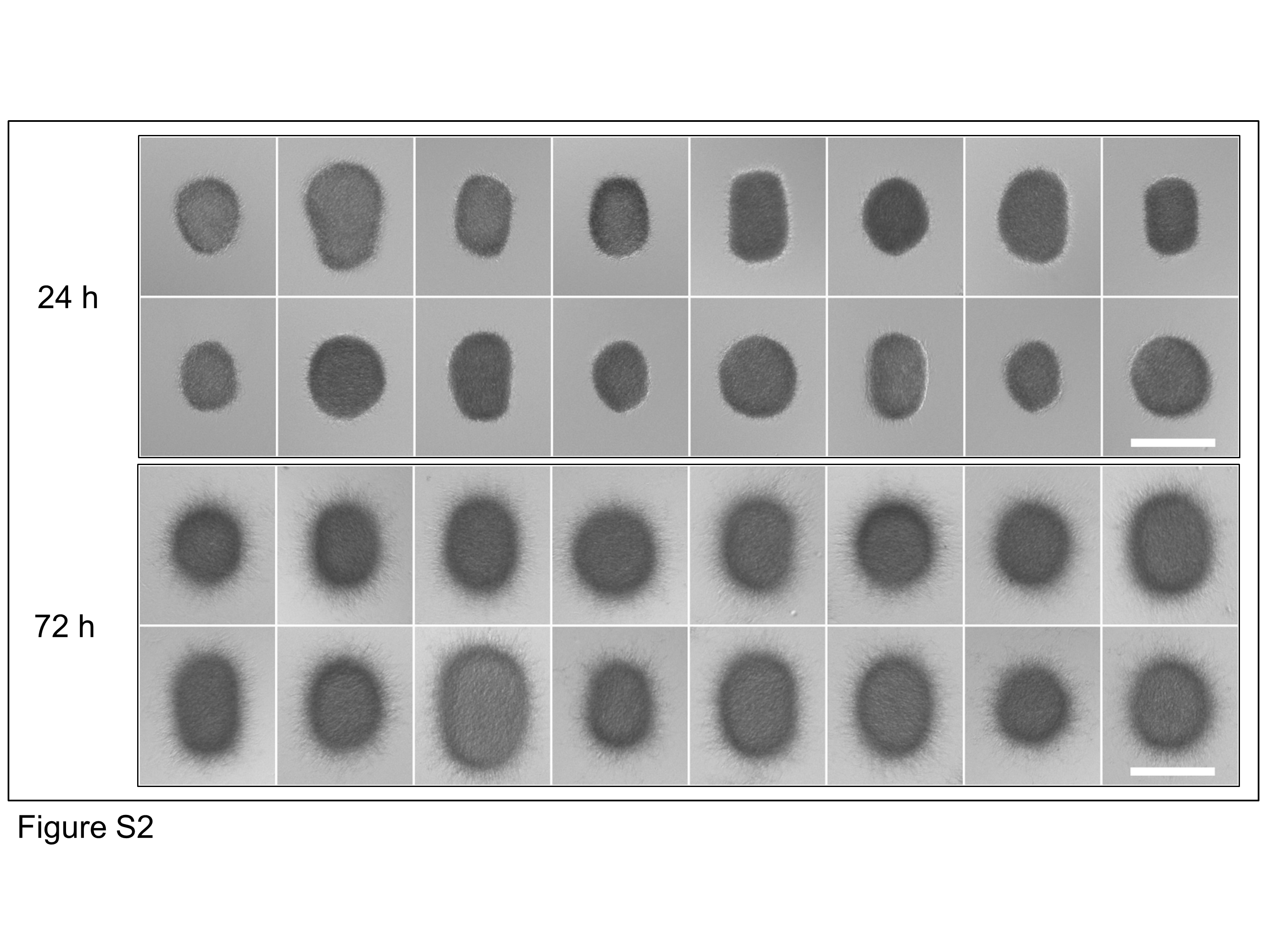

Supplement: FIGURE S2 — Morphological changes accompanying the aging of Streptomyces pellets. Collage of representative micrographs of pellets of S. lividans 66 in TSBS cultures after 24 (Top) and 72 h (Bottom) of growth. The scale bars represent 200 μm. [file Image_2.TIF]

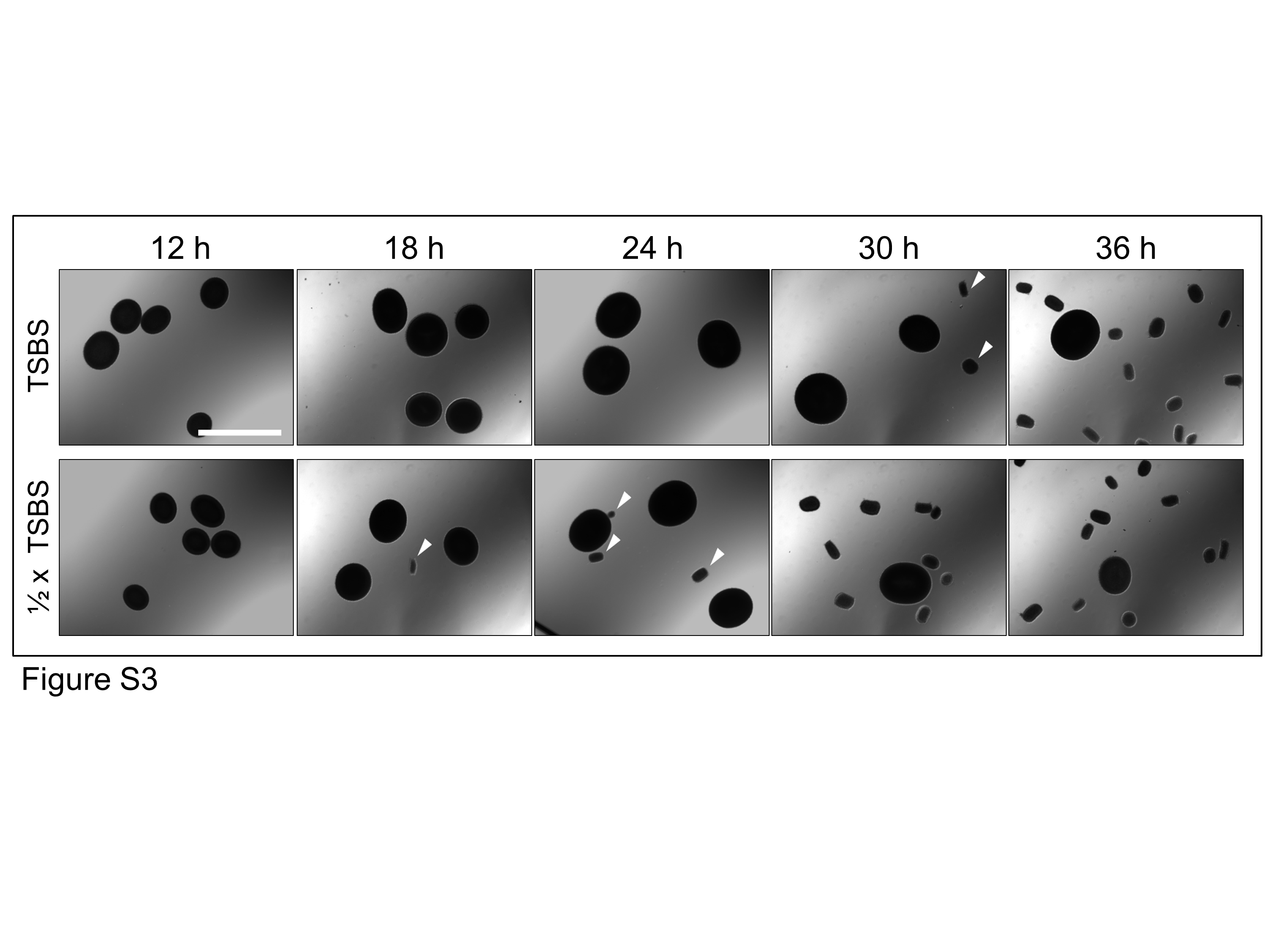

Supplement: FIGURE S3 — Fragmentation correlates with nutrient availability. Micrographs representing pellets of S. lividans 66 obtained by diluting seed cultures in fresh TSBS medium after 12, 18, 24, 30, and 36 h of growth. The seed cultures were prepared in normal TSBS (Top) or in ½x TSBS (Bottom) medium. Note that the small pellets, derived from outgrowing fragments, are observed at least 12 h earlier in ½x TSBS than in normal TSBS. The scale bar represents 1 mm. [file Image_3.TIF]

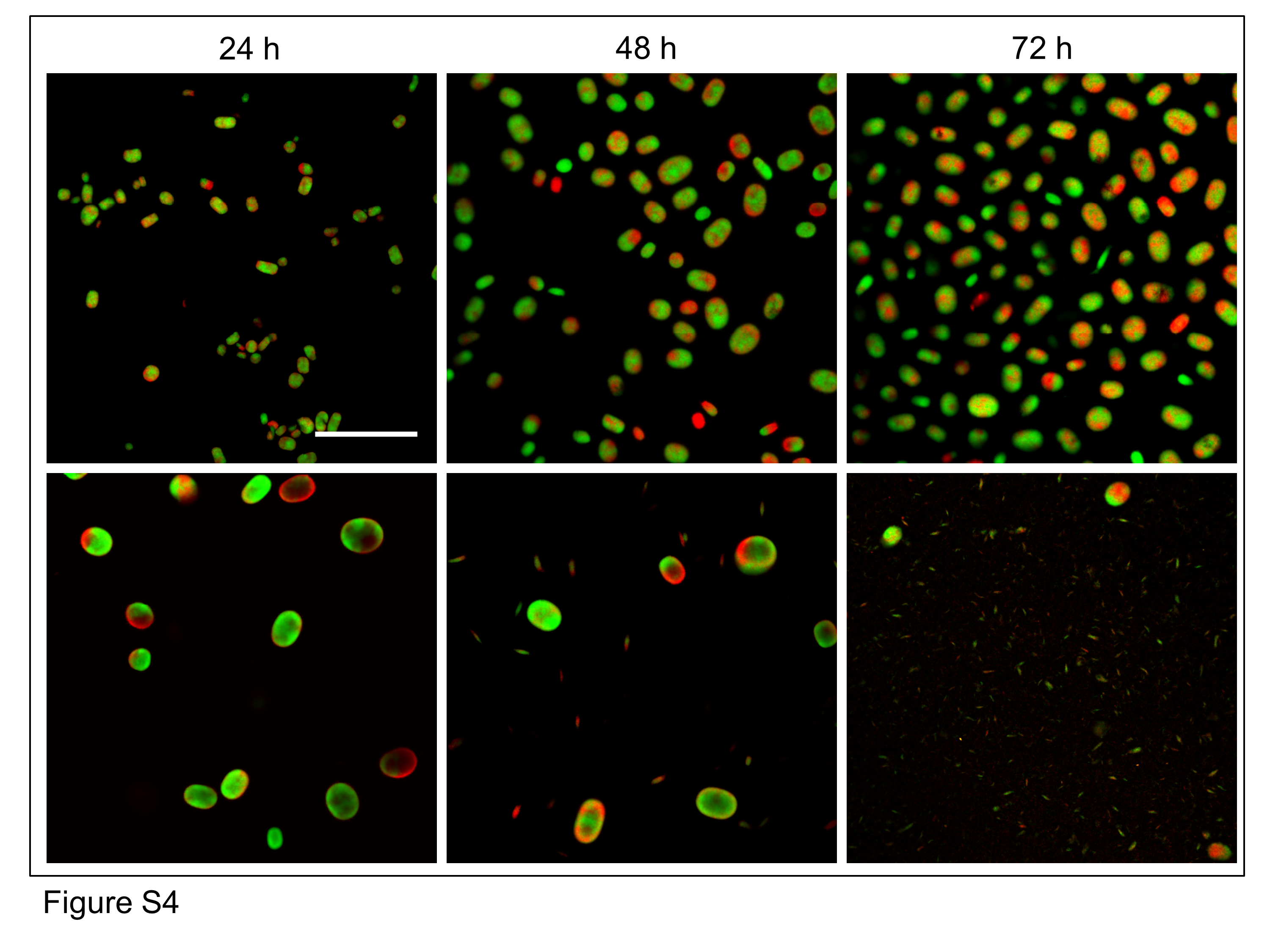

Supplement: FIGURE S4 — Morphological analysis of pellets of the fluorescent derivative strains of S. lividans in TSBS cultures. Seed cultures were prepared by co-culturing S. lividans strains constitutively expressing eGFP or mCherry in TSBS medium. The morphology of pellets in these seed cultures after 24 (Left), 48 (Middle) and 72 (Right) h of growth are shown in the top panels. The Bottom show micrographs of pellets following the transfer of 10 ml of the seed cultures in fresh TSBS medium and subsequent growth for 24 h. The scale bar represents 1 mm. [file Image_4.TIF]

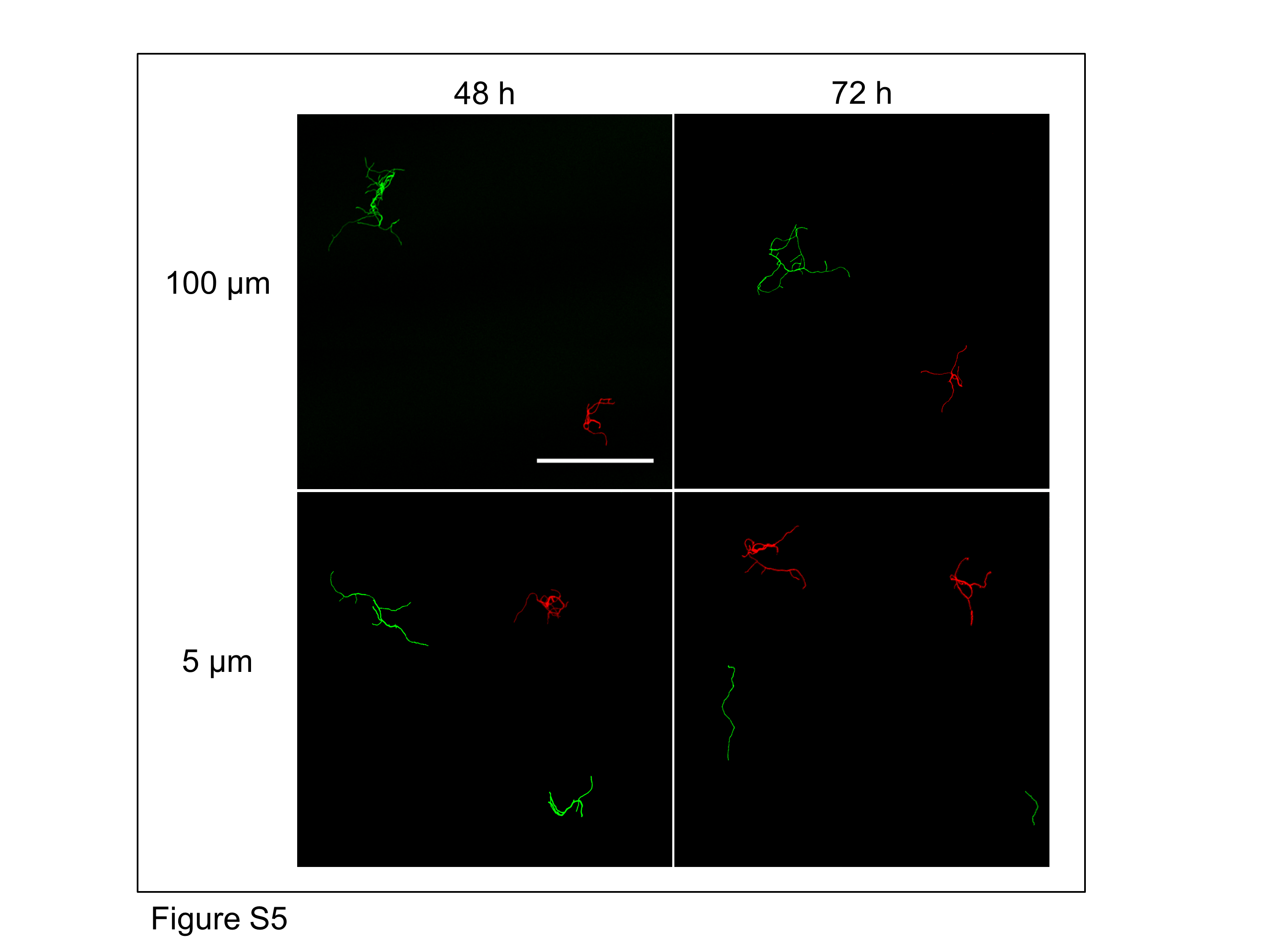

Supplement: FIGURE S5 — Visualization of detached fragments from pellets of co-cultured fluorescent S. lividans strains. Filtrates were obtained by the sequential filtering of TSBS cultures, which had been grown for 48 or 72 h, through cell strainers with a pore size of 100 (Top), 40 and 5 μm (Lower). Note that the detached fragments are either green or red fluorescent. The scale bar represents 100 μm. [file Image_5.TIF]

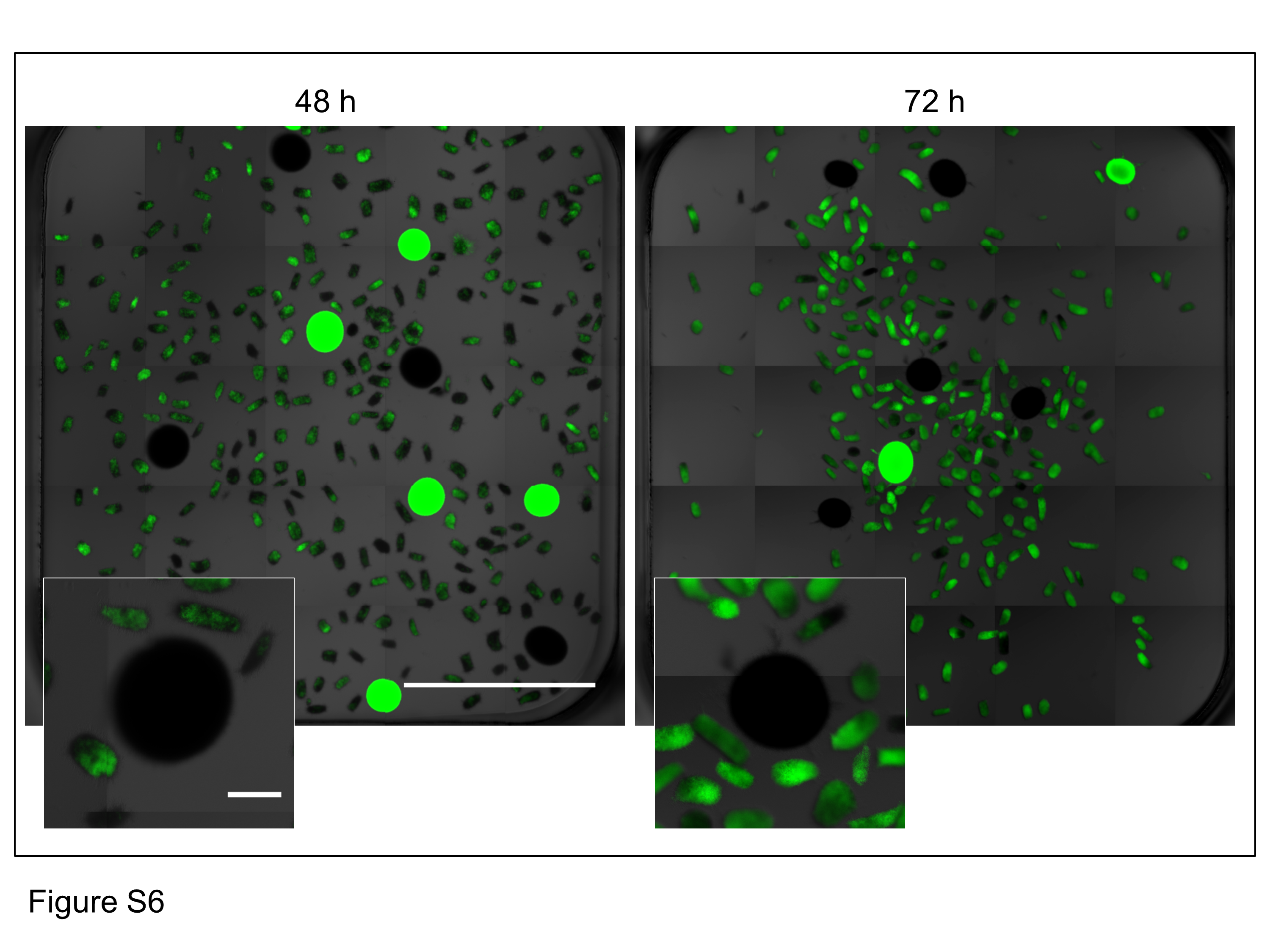

Supplement: FIGURE S6 — Large, fragmenting particles are inert to aggregation. Micrographs of pellets from co-cultures of the S. lividans wild-type strain and its green-fluorescent derivative, obtained by mixing separate cultures of both strains after 48 (Left) and 72 h (Right) of growth. The inlay shows that the large wild-type pellets remain non-fluorescent after the transfer, indicating that small mycelial fragments do not aggregate with these large particles. The scale bars represent 2 mm and 200 μm in the overview pictures and inlays, respectively. [file Image_6.TIF]
